# Personalized Interventional Management of Femoral Pseudoaneurysms of Iatrogenic and Traumatic Origin: Technical Aspects, Clinical Outcomes, and Risk-Adapted Treatment Selection

**DOI:** 10.3390/jpm16050239

**Published:** 2026-04-30

**Authors:** Antonio Borzelli, Francesco Giurazza, Luigi Basile, Fabio Corvino, Felice D’Antuono, Francesco Pane, Milena Coppola, Alessandro Punzi, Gianluca Cangiano, Antonio Corvino, Raffaella Niola

**Affiliations:** 1 Vascular and Interventional Radiology Department, Cardarelli Hospital, 80131 Napoli, Italy; antonio.borzelli@aocardarelli.it (A.B.); francesco.giurazza@aocardarelli.it (F.G.); fabio.corvino@aocardarelli.it (F.C.); felice.dantuono@aocardarelli.it (F.D.); francesco.pane@aocardarelli.it (F.P.); milena.coppola@aocardarelli.it (M.C.); alessandro.punzi@aocardarelli.it (A.P.); gianluca.cangiano@aocardarelli.it (G.C.); raffaella.niola@aocardarelli.it (R.N.); 2 Fatebenefratelli Hospital, 80123 Napoli, Italy; basileluigi92@gmail.com; 3 Medical, Movement and Wellbeing Sciences Department, University of Naples “Parthenope”, 80133 Napoli, Italy

**Keywords:** pseudoaneurysm, embolization, stent, stent graft, interventional radiology

## Abstract

**Background**: Femoral pseudoaneurysms are clinically heterogeneous, with substantial variability in anatomical features and patient-related bleeding risk. Standard treatment algorithms may be inadequate, particularly in patients receiving anticoagulation or presenting with altered coagulation profiles. A personalized, risk-adapted interventional strategy may optimize outcomes while preserving procedural safety. This study compares ultrasound-guided compression with endovascular and percutaneous therapies and evaluates the safety of minimally invasive approaches across different risk profiles to support individualized management. **Methods**: This single-center retrospective cohort study included 65 consecutive patients treated for femoral pseudoaneurysms between January 2019 and May 2025. Treatment modalities comprised ultrasound-guided compression, endovascular embolization (coils, covered stents, NBCA–Lipiodol), percutaneous glue injection, and hybrid approaches. Primary endpoints were technical and clinical success. Safety was assessed using pre- and post-procedural INR, platelet count, and hemoglobin levels. High-risk status was defined as ongoing anticoagulation or antiplatelet therapy, INR > 1.5, or platelet count <50 × 10^9^/L. **Results**: Endovascular and percutaneous approaches achieved significantly higher technical (100% vs. 68.5%, *p* = 0.006) and clinical success rates (100% vs. 77.8%, *p* = 0.009) compared with ultrasound-guided compression. In minimally invasive cohorts, INR and platelet counts remained stable after treatment, while hemoglobin showed an expected post-procedural decrease (*p* < 0.001). High-risk patients demonstrated technical success rates comparable to standard-risk patients, with no significant differences in laboratory trends. Favorable outcomes were observed across different embolic materials. **Conclusions**: Endovascular and percutaneous therapies provide superior effectiveness compared with ultrasound-guided compression while maintaining a reassuring safety profile, even in patients at increased bleeding risk. These findings support a personalized, patient-tailored interventional approach based on individual anatomical and clinical characteristics.

## 1. Introduction

Femoral pseudoaneurysms (FPAs) are pulsatile, blood-filled cavities that arise following disruption of the arterial wall and are contained by the surrounding periarterial tissues rather than by the native arterial layers. They most commonly occur as complications of arterial access procedures, although they may also develop after traumatic injury. If left untreated, FPAs may lead to clinically significant complications, including rupture, distal embolization, and limb ischemia, thereby warranting prompt diagnosis and appropriate management [1]. Duplex ultrasound represents the first-line diagnostic modality and typically demonstrates the characteristic “to-and-fro” Doppler waveform at the pseudoaneurysm neck, allowing for both confirmation of the diagnosis and initial anatomical assessment [2,3]. In cases of limited sonographic windows or complex anatomy, computed tomographic angiography can provide a more comprehensive delineation of vascular structures, while catheter angiography remains essential when an endovascular approach is planned, offering a detailed procedural roadmap [4]. Over the past decade, the management of FPAs has progressively shifted toward minimally invasive strategies. Ultrasound-guided compression (UGC) is widely available and non-invasive; however, it may be time-consuming, operator-dependent, and often uncomfortable for patients. Moreover, its effectiveness can be reduced in the presence of challenging anatomical features or ongoing antithrombotic therapy [4]. In contrast, ultrasound-guided thrombin injection (UGTI) has emerged as a cornerstone percutaneous technique, demonstrating high primary occlusion rates and a low incidence of complications across large clinical series [5,6]. The growing adoption of UGTI reflects a broader transition toward image-guided, minimally invasive treatments that prioritize both efficacy and patient comfort. In contemporary practice, optimal treatment selection increasingly relies on the integration of patient-specific and lesion-specific factors, reflecting a precision-oriented approach to interventional care. This paradigm emphasizes individualized procedural planning and tailored device selection, particularly in the setting of complex anatomy or high-risk clinical scenarios. Such an approach is consistent with established interventional radiology experience in customized percutaneous treatments across vascular and non-vascular domains, as well as with evolving concepts of precision imaging-guided interventions [7]. A broad and continuously expanding endovascular and percutaneous armamentarium now enables rapid and durable pseudoaneurysm exclusion while preserving arterial patency when necessary. Comparative evidence suggests that UGTI is generally more effective than UGC, particularly in terms of primary success rates [8], and large cohort studies further support its efficacy in both simple and complex FPAs [9,10]. Technical success is closely related to procedural factors, including thrombin formulation, accurate delivery, and meticulous ultrasound guidance to minimize the risk of distal embolization [11]. Additional real-world studies confirm the robustness of UGTI even in patients receiving antithrombotic therapy [4,12]. Beyond percutaneous thrombin injection, endovascular strategies such as coil embolization and covered stent-graft placement represent well-established alternatives, supported by both clinical overviews and consensus documents. These approaches achieve high technical and clinical success when carefully selected according to anatomical features and patient-specific risk profiles [13,14]. Predictors of UGC failure—such as wide pseudoaneurysm necks, short tracts, large sac size, and elevated body mass index—further underscore the importance of appropriate modality selection in current clinical practice [15]. Observational evidence supports an anatomy- and risk-adapted strategy when choosing among coils, N-butyl cyanoacrylate (NBCA), and stent-grafts, with the aim of balancing effective exclusion with preservation of arterial flow [16,17,18]. Comparative studies reinforce these treatment patterns, highlighting the role of individualized decision-making [19]. In addition, NBCA-based and adjunctive techniques have expanded the percutaneous toolkit, demonstrating favorable outcomes in the management of peripheral pseudoaneurysms [20,21,22]. This is complemented by broader clinical experience in post-access FPAs, including the identification of factors influencing treatment success and the use of adjunctive strategies such as balloon-assisted glue delivery in selected anatomical scenarios [23,24,25]. Despite these advances, direct comparisons between different minimally invasive strategies in real-world clinical settings remain limited, particularly in heterogeneous populations that include both iatrogenic and traumatic FPAs and in high-risk patient subgroups. Accordingly, we conducted a single-center study of consecutive patients with FPAs of iatrogenic and traumatic origin treated between January 2019 and May 2025. The aim of this study was to evaluate the outcomes of different management strategies for femoral pseudoaneurysms in a real-world clinical setting, with particular attention to risk-adapted, patient-tailored treatment selection.

## 2. Materials and Methods

### 2.1. Study Design and Setting

This was a single-center, retrospective cohort study conducted at Cardarelli Hospital, a tertiary referral hospital. All consecutive patients with femoral pseudoaneurysms treated between January 2019 and May 2025 were screened. Reporting followed the STROBE checklist for observational studies [7]. Statistical analysis was performed using IBM SPSS Statistics for Windows, version 21.0 (IBM Corp., Armonk, NY, USA).

### 2.2. Patients and Eligibility

Inclusion criteria: (1) femoral pseudoaneurysm confirmed by duplex ultrasound (Aplio a—Canon^®^ Medical Systems Corporation, Tokyo, Japan) and/or computer tomography (CT) (128-slice dual-energy SOMATOM CT scanner, Siemens Healthineers AG, Forchheim, Germany); (2) age ≥ 18 years; (3) treatment by one of the predefined strategies (UGC, endovascular, percutaneous, or hybrid). Exclusion criteria: (1) non-femoral pseudoaneurysms; (2) incomplete clinical or imaging documentation; (3) prior definitive treatment at an outside facility. A total of 65 patients met the eligibility criteria.

### 2.3. Definitions and Outcomes

Primary endpoints were technical and clinical success by modality. Technical success was immediate exclusion of the pseudoaneurysm sac/neck on intraprocedural angiography or ultrasound without unplanned therapy. Clinical success was durable exclusion without reintervention and symptom resolution at follow-up. Safety endpoints included periprocedural complications and laboratory trends (INR, platelets, hemoglobin) pre- versus post-procedure.

### 2.4. Risk Stratification

High-risk patients were defined as those receiving antiplatelet and/or anticoagulant therapy at the time of treatment, or having INR >1.5, or platelets <50 × 10^9^/L. Comparative analyses were performed between high-risk and non-high-risk groups.

### 2.5. Treatment Techniques

Patients were allocated to UGC, endovascular, percutaneous, or hybrid strategies based on anatomy, clinical status, and operator judgment. Endovascular techniques included coil embolization (Micronester Embolization Coil, Cook Medical/Cook Incorporated, Bloomington, IN, USA; Tornado Embolization Microcoil, Cook Medical/Cook Incorporated, Bloomington, IN, USA), covered stent-graft placement (GORE^®^ VIABAHN^®^, W. L. Gore & Associates, Inc., Flagstaff, AZ, USA), and NBCA–Lipiodol embolization (Glubran^®^ 2 cyanoacrylate glue, GEM S.r.l., Viareggio, Italy; Lipiodol^®^ Ultra Fluid, Guerbet, Paris, France); percutaneous therapy comprised thrombin (FLOSEAL Hemostatic Matrix, Baxter Healthcare Corporation, Deerfield, IL, USA) or glue injection under ultrasound guidance. Procedures were performed in an angiography suite (Artis zee Siemens Healthcare GmbH, Forchheim, Germany) or interventional ultrasound room by board-certified interventional radiologists. Device selection and access were individualized to lesion morphology and clinical priorities.

### 2.6. Imaging Assessment and Follow-Up

Baseline evaluation included duplex ultrasound in all patients and CT. Intraprocedural imaging consisted of angiography for endovascular cases and real-time ultrasound for percutaneous injections. Post-procedural assessment included immediate imaging confirmation of exclusion. Clinical and laboratory follow-up were obtained from the medical record; imaging follow-up (duplex ultrasound) was performed according to local practice.

### 2.7. Statistical Analysis

Continuous variables were summarized as mean ± standard deviation or median (interquartile range), as appropriate; categorical variables as counts and percentages. Group comparisons used Fisher’s exact for categorical variables and Mann–Whitney U, Wilcoxon signed-rank, or Kruskal–Wallis for continuous variables, as applicable. Two-sided *p*-values < 0.05 were considered statistically significant. Statistical reporting followed SAMPL and ICMJE guidance [26,27].

## 3. Results

### 3.1. Study Population

Sixty-five patients were included. Females accounted for 49.2% (*n* = 32) and males for 50.8% (*n* = 33); mean age was 65.1 ± 20.1 years (median 72). Patients were stratified according to baseline bleeding risk, with high-risk patients showing a mean age of 49.2 ± 20.2 years versus 66.1 ± 19.8 years in non-high-risk patients (*p* = 0.083). Baseline characteristics are summarized in Table 1.

### 3.2. Etiology and Lesion Characteristics

Etiologies included endovascular procedures in vascular radiology (48%), hemodynamics (18%), neurointerventional procedures (11%), orthopedic interventions (8%), and non-iatrogenic causes (8%). Lesion locations were the common femoral artery (39%), deep femoral artery (39%), and superficial femoral artery (22%), with right-sided involvement in 67% of cases and left-sided involvement in 32%. Mean pseudoaneurysm diameter was 23.24 ± 17.35 mm. This heterogeneity in etiology, anatomy, and lesion size reflects the need for individualized treatment selection. Etiology and lesion characteristics are detailed in Table 2.

### 3.3. Treatment Strategies and Devices

Ultrasound-guided compression (UGC) was used in 48% of cases, endovascular approaches in 51%, percutaneous treatments in 5%, and hybrid strategies in 3%. Embolic materials included coils (47.5%), NBCA–Lipiodol (22.5%), covered stents (7.5%), and vascular closure devices (5%) (ANGIO-SEAL^®^ VIP Vascular Closure Device, Terumo Medical Corporation, Somerset, NJ, USA), with combined material use (e.g., coils plus Onyx, Onyx Liquid Embolic System, Medtronic/Micro Therapeutics, Inc., Irvine, CA, USA) in 5% of cases. Treatment allocation and material selection were guided by lesion anatomy, access characteristics, and patient-specific clinical risk profiles. Allocation strategies and devices are summarized in Table 3. Totals may exceed 100% owing to staged or combined approaches.

### 3.4. Primary Outcomes

Endovascular and percutaneous therapies achieved significantly higher success rates compared with UGC, with technical success of 100% versus 68.5% (*p* = 0.006) and clinical success of 100% versus 77.8% (*p* = 0.009). These differences highlight the effectiveness of minimally invasive approaches when selected according to individual lesion and patient characteristics. Primary outcomes are reported in Table 4.

### 3.5. Laboratory Trends and Safety

In minimally invasive treatment cohorts, INR and platelet counts did not change significantly following the procedure (*p* = 0.970 and *p* = 0.626, respectively), whereas hemoglobin levels decreased as expected (*p* < 0.001). The stability of coagulation parameters supports the safety of a risk-adapted minimally invasive strategy. Laboratory trends are detailed in Table 5.

### 3.6. High-Risk Subgroup

Among high-risk patients, technical success was 100% and clinical success was 75%, with laboratory stability comparable to that observed in non-high-risk patients. These findings indicate that elevated baseline bleeding risk did not adversely affect technical feasibility when a personalized interventional approach was adopted. Subgroup outcomes and laboratory parameters are summarized in Table 5 and Table 6.

## 4. Discussion

**Figure 1:**
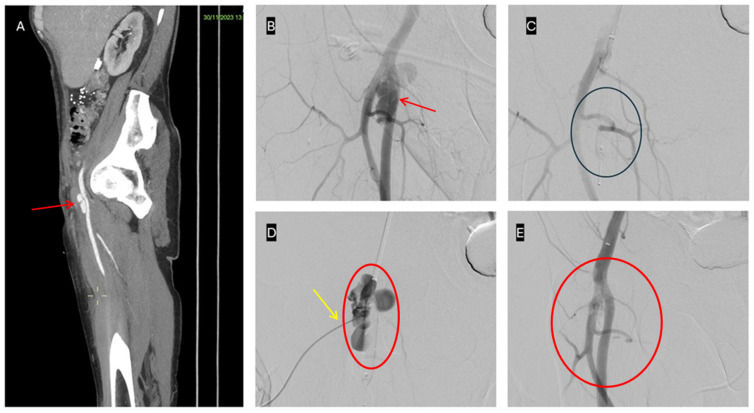
Hybrid endovascular and percutaneous treatment of a superficial femoral artery pseudoaneurysm. (**A**) Sagittal CT showing a multilobulated pseudoaneurysm of the right superficial femoral artery (red arrow). (**B**) Diagnostic angiography confirming the multilobulated pseudoaneurysm of the right superficial femoral artery (red arrow). (**C**) Endovascular balloon inflation across the neck for temporary exclusion of the pseudoaneurysm (blue circle). (**D**) Ultrasound- and fluoroscopy-guided direct NBCA–Lipiodol injection (yellow arrow) into the pseudoaneurysm sac (red circle). (**E**) Final diagnostic angiography confirming complete exclusion of the pseudoaneurysm (red circle).

**Figure 2:**
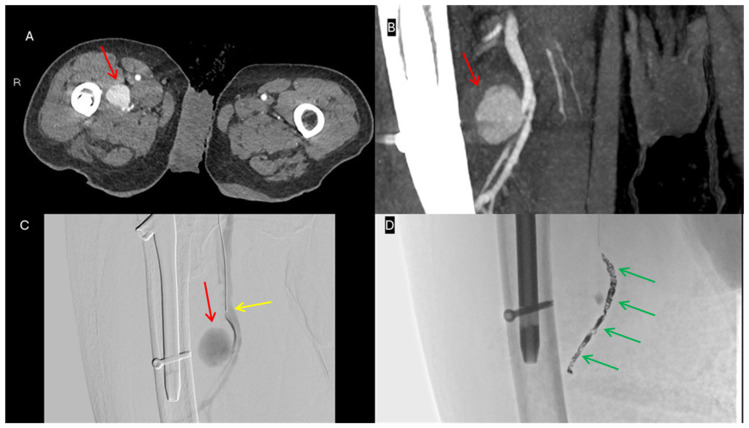
Endovascular coil embolization of a pseudoaneurysm of the right deep femoral artery. (**A**) Axial CT showing pseudoaneurysm arising from right deep femoral artery (red arrow). (**B**) Coronal CT reconstruction illustrating sac morphology (red arrow). (**C**) Selective angiography with microcatheter (yellow arrow) confirming the pseudoaneurysm (red arrow). (**D**) Coil embolization (green arrows), confirming complete exclusion of the pseudoaneurysm.

**Figure 3:**
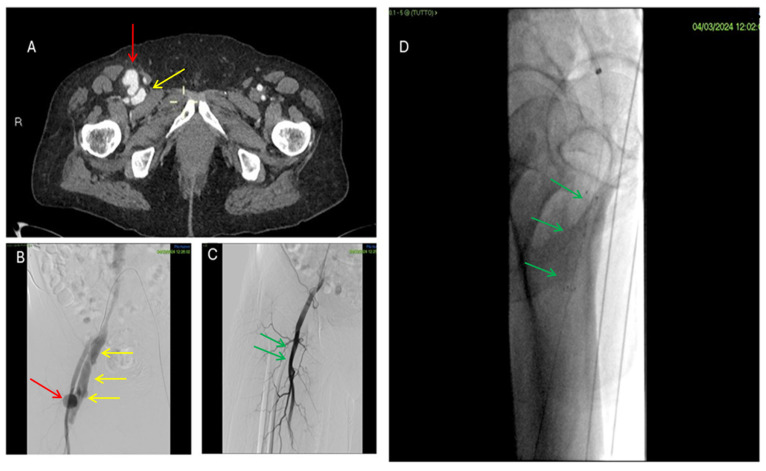
Endovascular covered stent exclusion of a rightsuperficial femoral artery pseudoaneurysm with an associated arteriovenous fistula. (**A**) Axial CT showing pseudoaneurysm (red arrow) and AV fistula (yellow arrow). (**B**) Diagnostic angiography showing the pseudoaneurysm (red arrow) and early venous opacification (yellow arrows). (**C**) Post-stenting (green arrows) diagnostic angiography confirming both pseudoaneurysm and fistula exclusion. (**D**) Deployment of the covered stent (green arrows).

**Figure 4:**
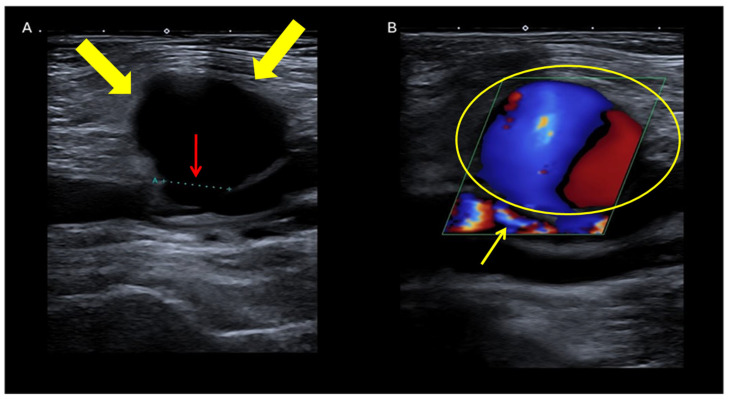
Ultrasonographic appearance of a femoral pseudoaneurysm. (**A**) B-mode US showing the pseudoaneurysm sac (yellow arrows) and its neck(red arrow). (**B**) Color Doppler US showing the characteristic “to-and-fro” waveform at the pseudoaneurysm neck (yellow arrow) and the bidirectional turbulent flow into the sac (yellow circle). Color Doppler coding: red and blue indicate arterial blood flow in opposite directions, toward and away from the transducer.

**Figure 5:**
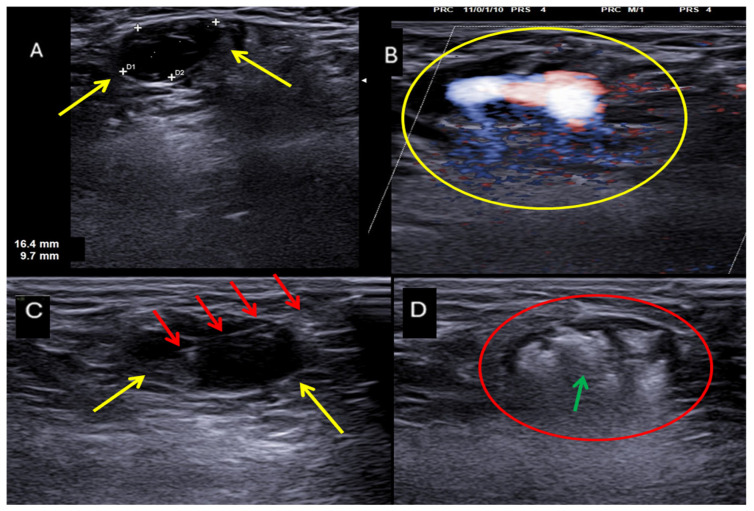
Percutaneous treatment of a femoral pseudoaneurysm by ultrasound-guided thrombin injection. (**A**) B-mode US showing the pseudoaneurysm sac (yellow arrows). (**B**) Color Doppler US showing bidirectional turbulent flow into the sac and its neck (yellow circle). (**C**) Ultrasound-guided puncture (red arrows) of the pseudoaneurysm sac (yellow arrows), which is anechoic and therefore patent. (**D**) B-mode US showing the pseudoaneurysm sac (red circle) completely filled with hyperechoic material after thrombin injection (green arrow).

**Figure 6:**
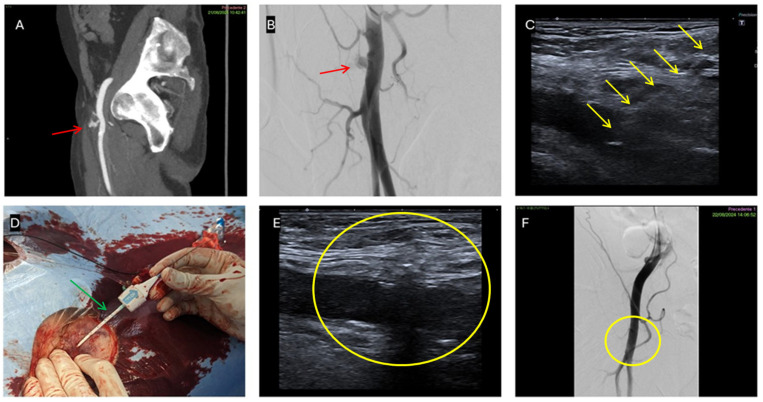
Percutaneous treatment with an Angio-Seal vascular closure device. (**A**) Axial CT of common femoral artery pseudoaneurysm (red arrow). (**B**) Diagnostic angiography confirming the pseudoaneurysm (red arrow). (**C**) Ultrasound-guided puncture and wire placement across the pseudoaneurysm neck into the common femoral artery lumen (yellow arrows). (**D**) Percutaneous Angio-Seal (green arrow) vascular closure device deployment. (**E**) Post-procedural Ultrasound confirmation of pseudoaneurysm’s exclusion (yellow circle). (**F**) Final diagnostic angiography confirming complete pseudoaneurysm’s exclusion (yellow circle).

In this single-center cohort, endovascular and percutaneous strategies—including combined approaches—demonstrated high technical and clinical success, particularly in patients selected based on lesion complexity and clinical risk profile, while ultrasound-guided compression remained effective in appropriately selected cases (Table 4). Although femoral pseudoaneurysm management has been established for decades, our study provides novel insights by reporting real-world outcomes of 65 consecutive patients treated with both traditional (UGC) and minimally invasive endovascular/percutaneous strategies. Importantly, we demonstrate that a patient- and lesion-tailored approach—including the use of multiple devices and materials guided by anatomical and risk-specific considerations—achieves high technical and clinical success, even in high-risk patients receiving anticoagulant or antiplatelet therapy. These findings highlight the clinical value of precision-oriented, individualized interventional strategies in optimizing efficacy and safety. These findings not only confirm the effectiveness of minimally invasive techniques but also support their safety profile in routine clinical practice. Beyond purely technical performance, our results reinforce the concept of a risk-adapted and patient-tailored interventional paradigm, in which treatment selection is not dictated by a rigid algorithm but rather guided by an integrated assessment of lesion-specific morphology and patient-specific clinical factors. In this setting, femoral pseudoaneurysm management represents a paradigmatic example in which individualized decision-making can directly translate into improved procedural efficacy and safety outcomes. Our findings are consistent with prior comparative studies favoring ultrasound-guided thrombin injection (UGTI) over compression therapy in iatrogenic femoral pseudoaneurysms [8], as well as with large clinical series demonstrating durable UGTI performance across both simple and complex pseudoaneurysm anatomies [9,10,11]. Importantly, these favorable outcomes have also been confirmed in patients receiving anticoagulant or antiplatelet therapy [4,12], a population that increasingly characterizes contemporary interventional practice. This aspect is particularly relevant within a precision medicine framework, as coagulation status and antithrombotic regimens are key determinants of both procedural risk and treatment feasibility. In cases where direct sac puncture is anatomically unfavorable—such as deep-seated lesions, wide-neck pseudoaneurysms, multilobulated morphology, or proximity to critical arterial branches—or when percutaneous access is associated with increased procedural risk, endovascular alternatives including coil embolization and covered stent-graft placement provide effective exclusion while preserving arterial patency. These techniques have consistently demonstrated high technical and clinical success in both practice overviews and guideline documents [13,14], supporting their role as integral components of a tailored therapeutic strategy rather than merely second-line rescue options. In parallel, well-established predictors of UGC failure, including wide necks, short tracts, larger sac diameters, and elevated body mass index, are frequently encountered in modern access-site complications and further justify the upfront selection of minimally invasive approaches in appropriately stratified patients [15]. This highlights the importance of early risk stratification in guiding treatment selection and optimizing procedural outcomes. Material selection represents an additional layer of personalization in the management of femoral pseudoaneurysms. Observational data support the choice between coils and N-butyl cyanoacrylate (NBCA) based on sac morphology, flow dynamics, and operator expertise [16,17,18,19], while multiple studies confirm the high effectiveness of UGTI and percutaneous glue embolization across peripheral pseudoaneurysm territories [20,21,22]. The integration of adjunctive techniques, such as balloon-assisted glue delivery, provides further technical refinement in challenging scenarios—particularly in wide-necked or multilobulated sacs—by improving embolic control and minimizing the risk of non-target embolization when performed by experienced operators [25] (Figure 1). In our center, percutaneous NBCA administration via direct puncture or transcatheter approaches achieved high technical and clinical success with only low-grade complications, even among patients receiving antithrombotic therapy [28]. Similar findings have been reported in the literature for both percutaneous and endovascular techniques, including coil embolization and covered stent placement, which remain reliable options across a range of anatomical settings (Figure 2 and Figure 3). Ultrasound evaluation plays a central role throughout the entire management pathway, from diagnosis to procedural planning and real-time guidance. By enabling detailed assessment of sac morphology, neck characteristics, and flow dynamics, ultrasound supports appropriate triage and facilitates selection of the most suitable technique on an individualized basis (Figure 4 and Figure 5) [23]. In selected cases, vascular closure devices may further assist in tract sealing while maintaining arterial patency, thereby expanding the range of available personalized interventional options (Figure 6) [24]. Taken together, these findings underscore the clinical value of a personalized interventional approach in femoral pseudoaneurysm management. Rather than applying a uniform treatment algorithm, modality and material selection can be tailored to anatomical complexity and patient-related risk factors, integrating variables such as coagulation status, ongoing antithrombotic therapy, and lesion characteristics into procedural decision-making. This patient-centered framework closely aligns with broader trends in precision medicine [7], highlighting how minimally invasive strategies can achieve high success rates without compromising safety when appropriately individualized. Femoral pseudoaneurysms are associated with a spectrum of potential complications, ranging from minor local effects to life-threatening events. Minor complications include local hematoma, pain, and skin necrosis [1,23]. More serious complications can include rupture with acute hemorrhage, distal embolization resulting in limb ischemia, and arteriovenous fistula formation, particularly in pseudoaneurysms arising from the superficial or deep femoral arteries [1,2,3,23]. Infection of the pseudoaneurysm sac, although rare, may further complicate management and necessitate urgent intervention [23,24]. The risk of such complications is influenced by lesion size, neck morphology, anticoagulation status, and timing of diagnosis [4,15]. In our cohort, careful anatomical assessment and risk-adapted selection of minimally invasive treatments—including ultrasound-guided compression, thrombin or glue injection, and endovascular approaches—allowed avoidance of major periprocedural complications, supporting the safety of a patient-tailored interventional strategy [5,6,7,22,25,28]. Despite these encouraging results, several limitations should be acknowledged. Firstly, the single-center, observational design may limit the generalizability of our findings, as outcomes are influenced by operator expertise, procedural volume, and institutional protocols. Secondly, the lack of long-term follow-up data limits the assessment of pseudoaneurysm recurrence and late complications. Future multicenter studies with larger patient populations and standardized treatment algorithms are warranted to validate these findings and further refine risk-adapted strategies. It is important to note that treatment allocation was not randomized but guided by clinical and anatomical considerations. Although a formal stratified comparison of baseline characteristics—such as lesion location, size, and neck morphology—was not performed, available data suggest that the groups were generally comparable. This non-randomized allocation may introduce selection bias, limiting the interpretability of direct comparisons between groups. In addition, future research may explore the role of emerging embolic materials, advanced imaging techniques, and decision-support tools to further enhance precision-oriented treatment planning. The integration of these innovations may contribute to the development of more standardized yet flexible algorithms that retain the benefits of individualized care. Nonetheless, our experience demonstrates that a tailored, anatomy- and risk-driven approach can optimize procedural efficacy, minimize complications, and expand the interventional radiologist’s therapeutic armamentarium for managing femoral pseudoaneurysms across diverse clinical scenarios. By systematically integrating lesion-specific characteristics, patient comorbidities, coagulation status, and antithrombotic therapy into procedural planning, this study exemplifies a precision-oriented, patient-tailored strategy, highlighting how individualized interventional radiology can maximize both safety and effectiveness in femoral pseudoaneurysm management.

## 5. Conclusions

Endovascular/percutaneous therapies provide superior effectiveness compared with ultrasound-guided compression for femoral pseudoaneurysms while maintaining a favorable safety profile—including in high-risk patients. These results support a personalized, risk-adapted interventional strategy, in which treatment modality and material selection are tailored to individual patient characteristics, lesion anatomy, and coagulation status. A pragmatic algorithm is justified: attempt UGC for anatomically suitable sacs; escalate to endovascular exclusion (coil, NBCA, or covered stent) for wide-neck, multiloculated, or recurrent lesions, with modality selection guided by both anatomical and patient-specific risk factors to maximize technical success and minimize complications.

## Figures and Tables

**Table 1:** Baseline Characteristics by Treatment Group.

Variable	UGC (*n* = 31)	Endo/Percutaneous (*n* = 34)	*p*-Value
Age, years (mean ± SD)	67.2 ± 18.5	63.1 ± 21.3	0.42
Female sex, *n* (%)	15 (48%)	17 (50%)	0.87
Hypertension, *n* (%)	11 (35%)	10 (29%)	0.61
Cardiovascular disease, *n* (%)	6 (19%)	5 (15%)	0.72
Diabetes mellitus, *n* (%)	4 (13%)	5 (15%)	0.83
Renal insufficiency, *n* (%)	3 (10%)	5 (15%)	0.54
Oncologic history, *n* (%)	3 (10%)	5 (15%)	0.54

Data are mean ± SD or *n* (%).

**Table 2:** Lesion Characteristics by Treatment Group.

Characteristic	UGC (*n* = 31)	Endo/Percutaneous (*n* = 34)	*p*-Value
Etiology—vascular radiology, *n* (%)	13 (42%)	18 (53%)	0.35
Etiology—hemodynamics, *n* (%)	6 (19%)	6 (18%)	0.92
Etiology—neurointerventional, *n* (%)	3 (10%)	4 (12%)	0.81
Etiology—orthopedic, *n* (%)	2 (6%)	3 (9%)	0.65
Etiology—non-iatrogenic, *n* (%)	2 (6%)	3 (9%)	0.65
Location—CFA, *n* (%)	12 (39%)	13 (38%)	0.94
Location—DFA, *n* (%)	12 (39%)	13 (38%)	0.94
Location—SFA, *n* (%)	7 (22%)	8 (24%)	0.85
Pseudoaneurysm diameter, mm (mean ± SD)	24.1 ± 16.8	22.5 ± 18.2	0.71

Values are % unless otherwise stated. Abbreviations: CFA = common femoral artery; DFA = deep femoral artery; SFA = superficial femoral artery.

**Table 3:** Treatment Strategies and Materials by Group.

Strategy/Device	UGC (*n* = 31)	Endo/Percutaneous (*n* = 34)
Ultrasound-guided compression	31 (100%)	0
Endovascular techniques	0	17 (50%)
Percutaneous techniques	0	2 (6%)
Hybrid endovascular–percutaneous	0	3 (9%)
Coils used	0	16 (47%)
NBCA–Lipiodol used	0	7 (21%)
Covered stent used	0	3 (9%)
Angio-Seal used	0	2 (6%)
Combined materials	0	2 (6%)

Share of cases (%). Abbreviations: UGC = ultrasound-guided compression; NBCA = n-butyl cyanoacrylate.

**Table 4:** Outcomes by Treatment Group.

Outcome	UGC (*n* = 31)	Endo/Percutaneous (*n* = 34)	*p*-Value
Technical success, *n* (%)	21 (68%)	34 (100%)	0.006
Clinical success, *n* (%)	24 (77%)	34 (100%)	0.009
Complications, *n* (%)	3 (10%)	2 (6%)	0.63

*p*-values from Fisher’s exact test (two-sided). Abbreviation: UGC = ultrasound-guided compression.

**Table 5:** Laboratory Trends (Minimally Invasive Cohorts).

Marker	Pre-Procedure (Mean ± SD)	Post-Procedure (Mean ± SD)	*p*-Value
INR	1.2 ± 0.3	1.2 ± 0.4	0.970
Platelets (×10^9^/L)	210 ± 65	208 ± 61	0.626
Hemoglobin (g/dL)	13.8 ± 1.9	12.9 ± 1.7	<0.001

*p*-values from Wilcoxon signed-rank tests. Direction indicates post-procedural change. Abbreviations: INR = international normalized ratio.

**Table 6:** High-Risk Subgroup Outcomes.

Outcome	High-Risk (*n* = 12)	Non-High-Risk (*n* = 53)
Technical success, *n* (%)	12 (100%)	43 (81%)
Clinical success, *n* (%)	9 (75%)	49 (92%)
INR/Platelets stability	Stable	Stable

High-risk defined as anticoagulation/antiplatelet therapy, INR > 1.5, or platelets <50 × 10^9^/L.

## Data Availability

The data supporting the findings of this retrospective study are not publicly available due to privacy and ethical restrictions, and are not shared in order to protect patient confidentiality.

## Abbreviations

The following abbreviations are used in this manuscript:

FPAs	Femoral pseudoaneurysms
UGC	Ultrasound-guided compression
UGTI	Ultrasound-guided thrombin injection
NBCA	N-butyl cyanoacrylate
CT	Computer tomography
